# A Retrospectively Diagnosed Case of IgG4-Related Tubulointerstitial Nephritis Showing Good Renal Outcome and Pathological Progress

**DOI:** 10.1155/2013/953214

**Published:** 2013-01-30

**Authors:** Qiong Wu, Raima Nakazawa, Hisae Tanaka, Masayuki Endoh, Masafumi Fukagawa

**Affiliations:** Department of Internal Medicine, School of Medicine, Tokai University, Kanagawa, Isehara 259-1193, Japan

## Abstract

A 74-year-old man was hospitalized for diabetic nephropathy evaluation and assessment of the effect of treatment on his tubulointerstitial nephritis (TIN). When he was 62 years old, he developed polyarthralgia and had superficial lymph node swelling, mildly increased serum creatinine concentration, hypergammaglobulinemia, hypocomplementemia, high serum IL-2R level, and positive titer of antinuclear antibody. Several tissues were biopsied. Mild chronic sialadenitis and reactive lymphadenitis were identified. Renal specimen showed mild glomerular ischemia, extensive storiform fibrosis, and abundant infiltrating monocytes and plasma cells. He was treated with oral prednisolone and cyclophosphamide. After the treatment, most of his clinical parameters quickly returned to within the reference range. However, he developed diabetes mellitus soon after steroid therapy. At the time of rebiopsy, a high level of serum IgG4 was detected. The second renal biopsy showed diabetic nephropathy without any tubulointerstitial damage. The first biopsied tissues were retrospectively investigated. Large numbers of IgG4-positive plasma cells were detected in the kidneys and lymph nodes. A retrospective diagnosis of IgG4-related TIN with lymph node involvement was made. In conclusion, this paper describes a retrospectively diagnosed case of IgG4-related TIN with lymph node involvement, showing good clinical and pathological prognosis.

## 1. Introduction

With the increase in reports of cases of IgG4-related diseases (IgG4-RDs) such as IgG4-related kidney disease (IgG4-RKD), autoimmune pancreatitis, sialadenitis, and retroperitoneal fibrosis [1–9], the All Japan IgG4 team has established comprehensive diagnostic criteria for IgG4-RD [1]. The critical parameters are serum IgG4 level and the quantity of tissue-infiltrating IgG4-positive cells, which are defined as >135 mg/dL and >10 IgG4-positive cells/high-power field (HPF) and/or >40% IgG4/IgG-positive cell ratio, respectively. IgG4-RKD mostly affects the tubulointerstitium and induces tubulointerstitial nephritis (TIN) [2, 3]. IgG4-related TIN differs from non-IgG4-related TIN in that it shows characteristic storiform fibrosis and massive IgG4-positive plasma cell infiltration. However, before the concept of IgG4-RD was established, some cases with the above features could not be classified as IgG4-RD. 

Here, we report a case that was diagnosed as TIN with interstitial fibrosis 12 years ago and was retrospectively diagnosed as IgG4-related TIN with lymph node involvement. Meaningfully, the storiform fibrosis and infiltrating cells disappeared in the second renal biopsy specimen. 

## 2. Case Report

**Figure 1:**
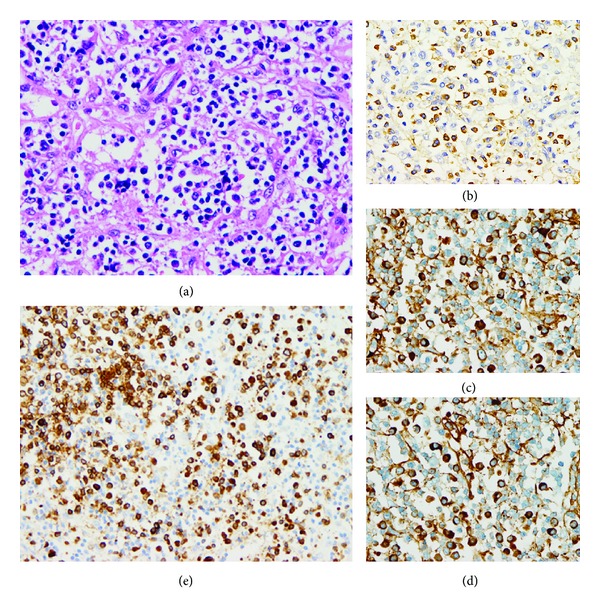
Lymph node biopsy tissue showing numerous plasma cells and fibrotic fibers ((a), HE staining, ×200). Immunohistological analysis revealed abundant CD79a-positive B cells ((b), ×200) and polyclonal plasma cells secreting kappa and lambda chains ((c) and (d), ×400). The existence of large numbers of IgG4-positive plasma cells was confirmed ((e), ×400).

**Figure 2:**
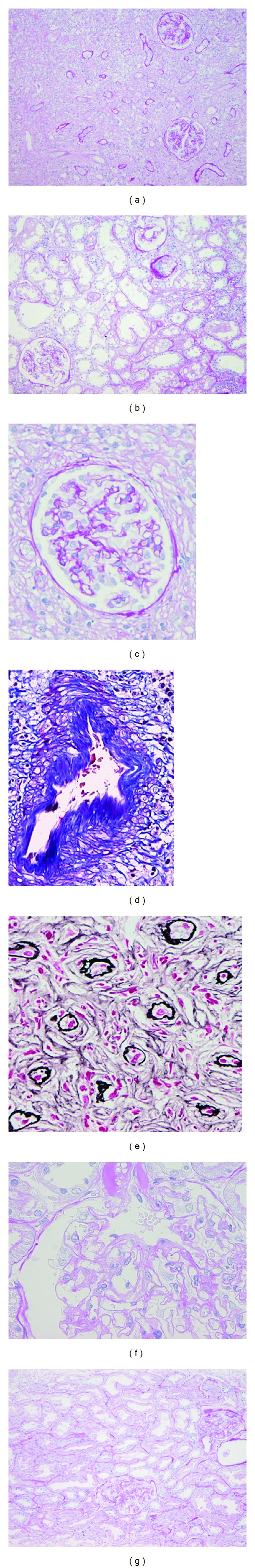
The first renal biopsy showed almost normal glomeruli, massively infiltrating cells, severe tubular atrophy, and abundant interstitial fibrosis ((a), PAS, ×100). Even in the almost normal area, mild cell infiltration and loss of tubular epithelia cells were observed ((b), PAS, ×100). Around the ischemic glomerulus ((c), PAS, ×200) and arteriole ((d), Masson, ×200), fibrotic fibers were observed. Most of these fibers appeared around tubules, and infiltrating cells formed a characteristic storiform pattern ((e), PAM, ×400). The second renal biopsy showed almost normal glomeruli with afferent arteriolar hyalinosis ((f), HE, ×400) and no tubulointerstitial damage ((g), PAS, ×400).

A 74-year-old Japanese man was hospitalized in 2008 for renal rebiopsy in order to diagnose diabetic nephropathy and to evaluate the effect of treatment on his TIN. Twelve years ago, when he was 62 years old, he was transferred to our hospital for polyarthralgia, especially on the shoulders and knees, and high gammaglobulinemia. His blood pressure was 170/82 mm Hg. Several lymph nodes ranging from 1 to 2 cm in diameter were palpated on the neck and axillary cavity, without tenderness. The abnormal clinical parameters are presented in Table 1. Serum IgE (<29.7 IU/mL) and urine beta2-microglobulin (BMG) (0.04 mg/L) (reference range <0.25 mg/L) were normal. Anti-dsDNA, anti-ssDNA, anti-DNA, anti-RNP, anti-SSA, and anti-SS-B antibodies; rheumatic factor; MPO-ANCA; PR3-ANCA; serum cryoglobulin; and urine Bence Jones protein were all negative. Serum immunoelectrophoresis showed chronic inflammatory changes and no monoclonal protein. Ophthalmological evaluation showed uveitis. Computed tomography (CT) scan showed low-density areas in both kidneys and multiple swollen lymph nodes about 10 mm in diameter in the submaxillary, subaural, collare, superior clavicle, mediastina, and axillary cavity regions. The sialogram was normal. Several organs were biopsied. Salivary gland biopsy showed mild chronic sialadenitis. Bone marrow biopsy showed marked hypocellular marrow in the needle-biopsied section and normocellular marrow in the clot section. Stomach biopsy showed minimal chronic gastritis. Axillary lymph node biopsy (Figures 1(a)–1(d)) showed reactive lymphadenitis. The immunohistochemical results were consistent with parafollicular hyperplasia with B-cell activation. A high number of CD79a-positive B cells and a polyclonal increase of plasma cells were identified. Open renal biopsy (Figures 2(a)–2(e)) showed that 2 of 30 glomeruli were sclerosed. The remaining glomeruli were almost normal or mildly ischemic. The tubulointerstitium showed remarkable tubular atrophy, tubular basement rupture, abundant mononuclear cell infiltration, and significant interstitial fibrosis. Among the infiltrating cells, multiple plasma cells were observed. Interstitial fibrosis presented a distinct storiform pattern, surrounding the glomeruli, tubules, arteries, veins, peritubular capillaries (PTCs), and infiltrating cell mass. The arteries showed no evidence of vasculitis, but their adventitia had disappeared and replaced by the surrounding fibrotic fibers. Mild tubulitis was observed. Approximately 10% of the total area appeared normal. However, the normal-appearing tissues were separated into smaller patches by infiltrating cells and fibrotic fibers. Mild cell infiltration and tubular cell loss were observed in these patches. Immunofluorescence showed no glomerular deposition of immunoglobulin and complements. A diagnosis of TIN with interstitial fibrosis was made. 

**Figure 3:**
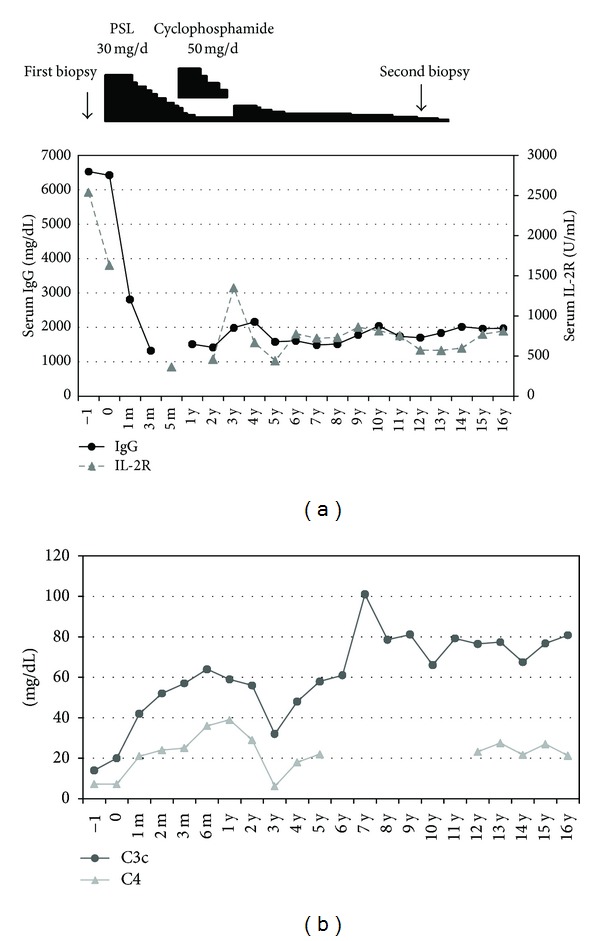
(a) Clinical treatment and serum IgG and IL-2R levels at followup. (b) Serum C3c and C4 levels at followup. PSL: prednisolone.

The patient was given oral prednisolone treatment starting with a dose of 30 mg/day, tapered 5 weeks later. Cyclophosphamide was started, at 50 mg/day, as a combination treatment after 8 months of prednisolone use. The cyclophosphamide dose was decreased 1 year later, and continued for a total of 2 years. Prednisolone was used for a total of 13.5 years (Figure 3(a)). After the treatment, the clinical parameters gradually returned to, or became close to normal. Within 1 month, urine N-acetyl-beta-D-glucosaminidase (NAG) decreased to 8.1 U/l and no longer exceeded the reference range. One month after the treatment, serum total protein and albumin returned to within the reference range (7.2 g/dL and 3.9 g/dL, resp.), although some fluctuations were observed during followup. Serum creatinine concentration decreased to 0.9 mg/dL and erythrocyte sedimentation rate (ESR) to 1 mm/h without subsequently increasing. Three months later, serum C1q binding immune complex decreased to <1.5 *μ*g/mL. Five months later, antinuclear antibody (ANA) became negative. Urine protein showed no obvious change during the followup period and remained negative. Hematuria was absent or in trace amount throughout the followup. Serum BMG fluctuated around the upper range value. Urine BMG increased from the 16th follow-up year.  Figure 3 shows the changes in serum IgG and IL-2R, and serum C3c and C4 during the follow-up period. The patient was followed for 16 years, and the final follow-up data are summarized in Table 1. 

Soon after the use of prednisolone, however, the patient developed diabetes mellitus. A corresponding treatment was given and the prednisolone was changed to cyclophosphamide, but his serum glucose was not well controlled. To estimate the effect of treatment and to survey the existence of diabetic nephropathy, a second renal biopsy was performed. At the time, his blood pressure was 124/66 mm Hg. Physical examination was normal. Laboratory findings showed a serum glucose level of 230 mg/dL and a urine glucose of 2+. The other parameters are shown in Table 1. Notably, the serum IgG4 concentration was 532 mg/dL (reference range 4.8–105 mg/dL). The needle-biopsied renal tissue was about 1.4 mm long and 1.0 mm wide with a cross-section area of 1.44 mm^2^. The tissue section for light microscopic examination contained 27 glomeruli, 2 of which were sclerosed globally. The other glomeruli showed mild capillary thickening and mild mesangial proliferation. Hyalinosis was observed in some afferents and arterioles (Figure 2(f)). No tubular atrophy and interstitial cell infiltration and fibrosis were observed (Figure 2(g)). Immunofluorescence showed a smooth, interrupted, and linear IgG deposition in the capillary loops. These findings were compatible with diabetic nephropathy. 

**Figure 4:**
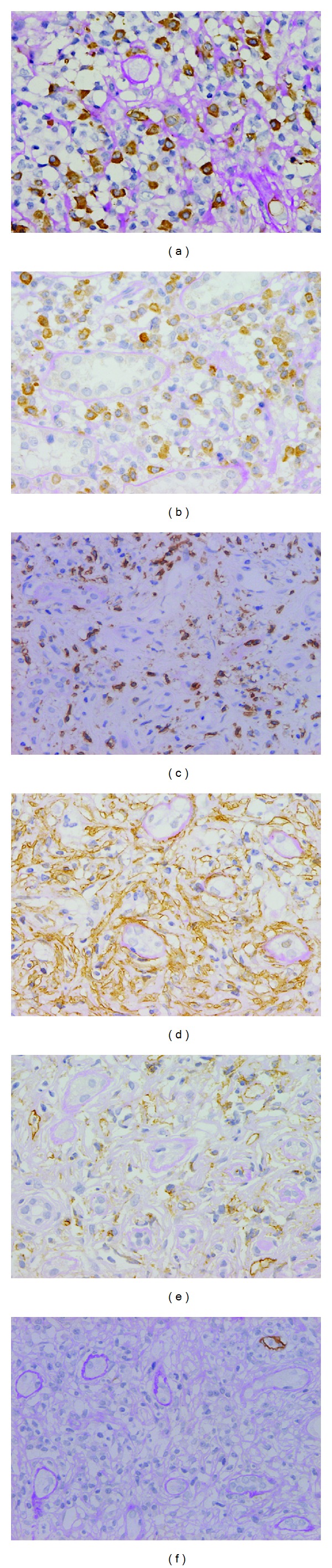
Retrospective immunostaining of the first renal biopsy tissue revealed high numbers of IgG4-positive plasma cells in the interstitium ((a), ×400). Many CD79a-positive B cells ((b), ×400), CD45RO-positive T cells ((c), ×200), and alpha-SMA-positive activated fibroblasts were also observed in the interstitium ((d), ×400). Large numbers of CD31-positive PTC ((e), ×400) and few D2-40-positive lymphatic vessels ((f), ×200) were identified.

As a retrospective investigation, the first biopsy tissues were subjected to IgG subclass immunostaining with monoclonal antibodies. Salivary gland specimen showed 5-6 IgG4-positive plasma cells/HPF, about 30% of IgG4/IgG-positive cell ratio, and no storiform fibrosis. Lymph node biopsy specimen showed more than 100 IgG4-positive plasma cells/HPF and storiform-like fibrosis (Figure 1(e)). Renal specimen showed many IgG-positive cells in the interstitium, mainly IgG4-positive plasma cells (Figure 4(a)). Other cell marker studies revealed a high number of activated B cells (CD79a positive), activated T cells (CD45RO positive), and activated fibroblasts (myofibroblasts, alpha-SMA positive) in the interstitium (Figures 4(b)–4(d)). CD31-positive PTCs were present in large numbers and distributed evenly (Figure 4(e)). In contrast, only a few D2-40-positive lymphatic vessels were observed around the arterial region and in the infiltrating cell mass. Almost no lymphatic vessels were found in the diffuse cell infiltration and fibrotic areas (Figure 4(f)). The lymphatic vessel density (lymphatic vessel number/interstitial area) was 3.9/mm^2^. A retrospective diagnosis of IgG4-related TIN with lymph node involvement was made. 

## 3. Discussion

Reports about cases of IgG4-RD have been increasing recently. The All Japan IgG4 team collects all such cases and has defined the concept of this novel entity. By its definition, IgG4-RD is characterized by organ enlargement or nodular/hyperplastic lesions in various organs, concurrently or metachronously, owing to marked infiltration of lymphocytes and IgG4-positive plasma cells, as well as fibrosis of unknown etiology [1]. IgG4-RKD is a type of IgG4-RD mainly affecting the kidney. Its diagnostic criteria have been discussed by many researchers [4] and were finally established by the All Japan IgG4 team [1, 3]. The present case demonstrated mild proteinuria, increased serum BMG, increased urine NAG, decreased renal function, elevated serum IgG, and hypocomplementemia at the first renal biopsy. CT scan confirmed the presence of low-density lesions in both kidneys. Renal biopsy showed massive IgG4-positive plasma cell infiltration and characteristic interstitial fibrosis. However, the serum level of IgG4 was not measured at that time because of unawareness of the existence of IgG4-RD. Nevertheless, the high level of serum IgG4 at the second renal biopsy could indicate the same high level at the first biopsy. IgG4-RKD generally affects the tubulointerstitium, referred to as IgG4-related TIN. The discriminating features of IgG4-related TIN from non-IgG4-related TIN are the numerous IgG4-positive plasma cells and the characteristic special fibrosis in the interstitium [2, 3, 5]. This special fibrosis is named storiform fibrosis [6] or bird eye pattern fibrosis [7] and shows high specificity and sensitivity in the diagnosis of IgG4-related TIN [4, 5]. In the present case, such fibrosis was abundantly present in the renal interstitium, which confirms the diagnosis of IgG4-related TIN. Additionally, the present case had a high titer of ANA but no evidence of systemic lupus erythematosus, which is considered another characteristic of IgG4-RD. Although not considered essential for diagnosis, many IgG4-RD patients have multiform autoantibodies without evidence of systemic diseases [10]. 

Most IgG4-RD patients show multiorgan involvement. The present patient also showed multiorgan symptoms involving the kidneys and lymph nodes. However, the involvement of the salivary gland, which is reported to be an organ susceptible to IgG4-related TIN [2, 7], could not be determined in the present case. Nevertheless, infiltrating IgG4-positive cells was observed in the biopsy specimen, but failed to reach the diagnostic criteria. Thus, early diagnosis and treatment of IgG4-RD is important in order to avoid the involvement of more organs. 

IgG4-RD always shows good response to treatment, mainly steroids [4, 7, 8]. Tsubata et al. [8] reported such a case of IgG4-related TIN. The patient's clinical parameters reached stable levels after 3 months of steroid treatment. The present case also showed a noticeable response to steroid treatment. The clinical parameters gradually returned to the reference range from 1 month after the treatment was started. The long-term stable value of urine NAG and serum creatinine indicated a benign course of renal recovery. The normalization of ESR, decrease of immune complex, increase of complement level, and disappearance of autoantibody revealed immune improvement. IgG4-related TIN is reported to have the feature of a clear margin between normal and damaged tissues [7], which is demonstrated by the present case. It is worth mentioning that the “almost normal” areas were not completely normal because they showed mild cell infiltration and tubular epithelial cell damage. In the re-biopsied renal tissue with an area of 1.44 m^2^, no cell infiltration and fibrosis was observed. Moreover, the pericapsular and perivascular fibrosis identified at the first biopsy was not detected at the second biopsy. All the above results suggest clinical and pathologic improvement in this case. 

Fibrosis is considered a histopathological indicator of poor prognosis [11–13]. Early steroid treatment before interstitial fibrosis develops is recommended in acute interstitial nephritis and other glomerulonephritis; otherwise, the patient will be unresponsive to treatment [11, 12]. T cells are common infiltrating cells in renal diseases, which are known to contribute to renal fibrosis and renal dysfunction [12, 13]. Lymphatic proliferation is also considered to contribute to tubulointerstitial fibrosis [13]; however, B cells and myofibroblasts have not been proven to have an association with fibrosis [12]. The present case exhibited massive interstitial fibrosis but good response to steroid treatment, resulting in the complete disappearance of the fibrosis. The disappearance of the fibrosis may have been responsible for the present patient's long-term stable renal function. The functions of infiltrating cells, renal intrinsic cells as well as the vascular system in fibrosis development and fibrotic fiber absorption are not known. Clinical followup and renal rebiopsy seem important in revealing the mechanism of IgG4-RD. 

In conclusion, this is a retrospectively diagnosed case of IgG4-related TIN with lymph node involvement that showed good clinical and pathological prognosis by steroid and immunosuppressant treatment. 

## Figures and Tables

**Table 1:** Clinical parameters at the first and second renal biopsy and at the final followup.

	Reference range	First biopsy	Second biopsy	Final followup
Hb (g/dL)	13.5–17.5	10.3	13.8	13.9
Serum TP (g/dL)	6.5–8.0	10.5	7.2	8.1
Serum Alb (g/dL)	3.9–4.8	3.2	3.8	3.9
Serum Cr (mg/dL)	0.5–1.1	1.3	0.9	1.01
ESR (mm/h)	0–15	127	ND	ND
Serum IgG (mg/dL)	870–1700	6530	1599	1974
Serum IgG4 (mg/dL)	4.8–105	ND	532	466
Serum IgA (mg/dL)	110–350	92	138	190
Serum IgM (mg/dL)	30–180	86	119	147
CH50 (U/mL)	25.0–48.0	<10.0	49	ND
Serum C3c (mg/dL)	85–160	18	68	80.8
Serum C4 (mg/dL)	15–40	<7.2	23.2	21.3
Serum C1qIC (*μ*g/mL)	<3.0	34.7	ND	ND
Serum IL-2R (U/mL)	220–530	2540	624	811
Serum BMG (mg/L)	0.5–1.7	5.15	1.88	ND
Urine BMG (mg/L)	<0.25	0.04	0.15	0.17
ANA	Negative	1280-fold	ND	ND
Urine protein (g/24 h)	<0.05	0.07	0.07	ND
Urine NAG (U/L)	<11.0	27.7	5.8	ND

Hb: hemoglobin; TP: total protein; Alb: albumin; Cr: creatinine; ESR: erythrocyte sedimentation rate; CH50: total serum hemolytic activity; C3c: complement 3c; C4: complement 4; C1qIC: C1q binding immune complex; BMG: beta2-microglobulin; ANA: antinuclear antibody; NAG: N-acetyl-beta-d-glucosaminidase; ND: not done.
